# Retailer opinions about and compliance with family smoking prevention and tobacco control act point of sale provisions: a survey of tobacco retailers

**DOI:** 10.1186/s12889-015-2231-2

**Published:** 2015-09-11

**Authors:** Shyanika W. Rose, Sherry L. Emery, Susan Ennett, Heathe Luz McNaughton Reyes, John C. Scott, Kurt M. Ribisl

**Affiliations:** Department of Health Behavior, Gillings School of Global Public Health, The University of North Carolina at Chapel Hill, Chapel Hill, NC USA; Lineberger Comprehensive Cancer Center, School of Medicine, University of North Carolina at Chapel Hill, Chapel Hill, NC USA; Health Media Collaboratory, Institute for Health Research and Policy, University of Illinois at Chicago, Chicago, IL USA; Department of Public Policy, University of North Carolina at Chapel Hill, Chapel Hill, NC USA; Schroeder Institute for Tobacco Research and Policy Studies at Truth Initiative, Washington, DC USA

## Abstract

**Background:**

The objectives of this study were to document retailer opinions about tobacco control policy at the point of sale (POS) and link these opinions with store level compliance with sales and marketing provisions of the Tobacco Control Act.

**Methods:**

This study conducted interviews of 252 tobacco retailers in three counties in North Carolina and linked their opinions with in-person observational audit data of their stores’ compliance with POS policies. We conducted analyses examining retailer factors associated with noncompliance using Generalized Estimating Equations (GEE) controlling for individual, store, neighborhood, and county factors.

**Results:**

Over 90 % of retailers support minors’ access provisions and a large minority (over 40 %) support graphic warnings and promotion bans. Low levels of support were found for a potential ban on menthol cigarettes (17 %). Store noncompliance with tobacco control policies was associated with both more reported retailer barriers to compliance and less support for POS policies. Awareness of and source of information about tobacco control regulations were not associated with compliance when accounting for neighborhood and county characteristics.

**Conclusions:**

Retailers expressed some support for a wide range of POS policies. Advocates and government agencies tasked with enforcement can work with retailers as stakeholders to enhance support, mitigate barriers, and promote compliance with tobacco control efforts at the point of sale.

## Background

In 2009, the Family Smoking Prevention and Tobacco Control Act (“Tobacco Control Act”) (Public Law 111–31) instituted new sales and marketing provisions at the point of sale (POS). Public policy theory suggests that the extent of policy implementation and compliance with new policy rests largely with ‘street level bureaucrats’ – implementers on the ground (in this case, tobacco retailers) [1]. Currently, tobacco retailers are often viewed as tobacco industry allies because their economic self-interest is tied to tobacco sales [2]. Convenience store associations have also served as front groups for the industry to block or blunt the effects of POS policy [2, 3]. However, a number of studies suggest that the majority of tobacco retailers are compliant with Tobacco Control Act POS sales and marketing provisions [4–6]. For example, two studies in Ohio examined the compliance of retailers with four Tobacco Control Act POS provisions, finding violation rates under 10 % [5, 6]. In 2012, the US Food and Drug Administration (FDA) conducted compliance checks in 37 states and the District of Colombia and issued warning letters or civil penalty letters to retailers for violations of sales and marketing restrictions (not including sales to minors) in only 1 % of the checks [7]. Our prior study in North Carolina identified a 15.7 % violation rate of any of 12 provisions of the Tobacco Control Act [4]. Taken together these studies suggest that it may be possible to engage tobacco retailers as *stakeholders* in tobacco control efforts, rather than adversaries.

Engaging with tobacco retailers in order to ensure implementation of Tobacco Control Act policies requires an understanding of the factors associated with retailer compliance. However, to date, little research has examined retailer characteristics and opinions that may be associated with compliance practices. The current study sought to understand how retailer opinions may be associated with compliance through interviews with 252 retailers whose stores had been audited for compliance with Tobacco Control Act POS provisions [4]. We assess four constructs related to Mazmanian and Sabatier’s Framework of Analysis for Policy Implementation [8, 9]: (1) retailer barriers to complying with regulations, (2) awareness of policy, (3) source of information about policies; and (4) retailer support for policies. Each of these constructs was then examined in relation to retailer compliance with Tobacco Control Act POS provisions.

## Methods

### Data source and study population

**Fig. 1:**
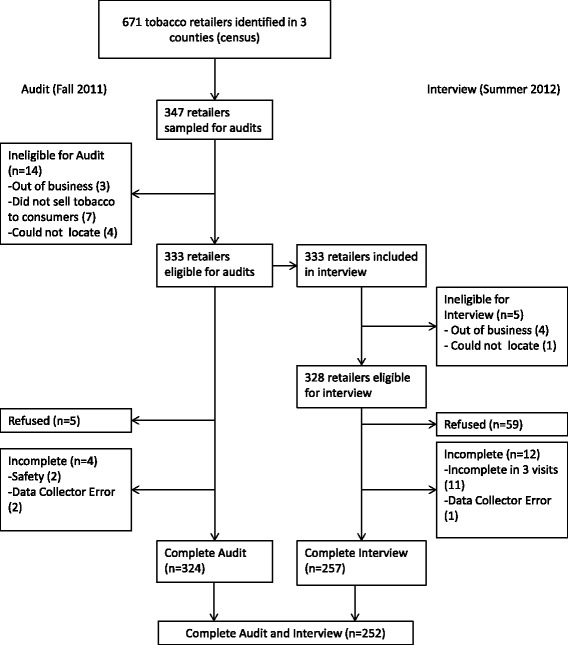
Study sample response diagram

This study linked interview data from retailers to data on store compliance with POS provisions as measured by store audits conducted by the research team (*Healthy Stores, Healthy Communities*) [4, 10]. We selected the three counties, Buncombe, Durham, and New Hanover, to represent distinct geographic regions of North Carolina (mountain, central, and coastal); all have a high cancer burden. Figure 1 shows the response for each data collection activity for this study. We identified all tobacco retailers within these three counties (*n* = 671) in Summer/Fall 2011 through driving all primary and secondary roads. In Fall 2011, we conducted in-person store audits of 347 retailers selected through stratified random sampling proportionate to the number of retailers in each county. Of these, we completed 324 audits; 14 stores were ineligible; 5 stores refused; and 4 stores were incomplete for safety or other reasons.

We then conducted retailer interviews in Summer 2012. Of the 324 stores with audit data, 4 stores were ineligible (out of business or could not be located) and 56 retailers refused to complete the interview. Interviews were incomplete in 12 stores – 1 store due to data collector error and 11 where the interview could not be completed in 3 attempts. For the interview, we achieved a response rate of 78 % of stores. The final sample of stores with both interview and audit data was 252.

### Respondent eligibility criteria

Respondents in the store were eligible if they were the owner, on-site manager/assistant manager, or store clerk of an audited tobacco retail outlet. If multiple potential respondents were in the store, we interviewed the “highest” ranking participant. Eligible respondents needed to speak English. Only one respondent, who also refused the interview, did not speak English. Respondents received a $20 gift card to a chain store as an incentive.

### Data collection and measures

We conducted all retailer audits electronically using data collection forms programmed in Pendragon on an iPod touch. The University of North Carolina at Chapel Hill Public Health–Nursing institutional review board determined that the audit did not constitute human subjects research and did not require approval.

For the interview, we piloted the questionnaire at 6 retailers in a county not selected for the study. Data collectors were trained in a half-day session on study procedures and were certified in using the interview instrument which was programmed in Qualtrics for use on an iPad. The interview was approved by the UNC–CH Public Health–Nursing institutional review board (Study Number #12-0548).

#### Compliance measure

The primary measure of compliance was from the audit. We assessed compliance with 12 POS provisions of the Tobacco Control Act implemented at the time of the store audit [4]. A store was non-compliant if there were any: (1) sale of flavored cigarettes, (2) “light” or “low tar” labeled cigarettes, (3) loose cigarettes, (4) loose smokeless tobacco, (5) branded non-tobacco products, (6) self-service of cigarettes or smokeless tobacco, (7) tobacco vending machines, (8) audio advertisements with sound effects, (9) video advertisements with sound effects, music, or color, (10) gifts given with cigarette or smokeless tobacco purchase, (11) availability of promotions offering gifts with proof of purchase (e.g., tobacco ‘reward’ programs), or (12) promotion of tobacco brand name event sponsorship.

#### Retailer opinions

Based on the Mazmanian and Sabatier framework, we used three measures focused on factors that influence the extent of change needed to comply with regulations: (1) Awareness, (2) Source of information, and (3) Barriers to Compliance. These factors are relevant because, first, if retailers are unaware of regulations, they may be unable to comply. Second, their source of information about regulations may influence the extent to which they receive timely and accurate information about compliance requirements. Finally, retailers may find it difficult to comply with regulations because of structural or logistic barriers.

*Awareness* was measured as whether retailers were aware of the Tobacco Control Act as a dichotomous item from from the 2009 International Tobacco Control US survey [11].

*Source of information* was measured through a series of yes/no/does not apply questions asking about usual source of information about tobacco control regulations. We assessed nine different sources including both formal sources (government, tobacco industry, corporate, boss/manager, and trade associations) and informal sources (media, family and friends, customers, and other retailers). Respondents could indicate multiple ‘usual sources.’ Respondents could also note if a source was not applicable to them. From these variables, we created a dichotomous variable of whether a retailer cited any formal source of information.

*Barriers to compliance* was assessed through four items measured on a five-point Likert scale from strongly disagree to strongly agree. Items were coded so that higher values indicated stronger agreement with barriers. Barriers items (i.e., hurts my business, too costly, takes too much time, too hard to redo displays/shelves) were only asked of owners and managers (*n* = 165), and not of clerks.

We assessed *level of support* for POS regulations by retailers through 10 items measured on a five-point Likert scale from strongly disagree to strongly agree. These items were drawn from or adapted from multiple sources [11–18]. Items were scored so that higher scores represented more support for POS regulations. We assessed support for POS provisions that had been enacted such as a ban on flavored cigarettes, and those delayed by litigation including graphic warning labels and black and white tobacco advertisements. This scale had two items addressing each of five different aspects of the provisions:

- bans on flavored and menthol cigarettes (product),
- graphic warning labels on packs and ads (counter advertising),
- black and white text ads and packaging (advertising and labeling),
- bans on gifts with tobacco sale and branded non-tobacco items (promotion),
- fines for retailers that sold tobacco to minors and increased fines for repeat sales (minor’s access).

#### Controls

We used control variables at the individual, store, neighborhood, and county levels as factors associated with either compliance, other tobacco marketing disparities, or with support for policy in prior studies.

##### Individual

Individual level variables included respondent *current smoking status* (everyday/some days vs. not at all) and *respondent role* (owner, manager, or clerk).

##### Store

We controlled for *store proximity to school* measured as a dichotomous variable of whether the store is within 1000 ft of a public school. We categorized *store type* (e.g., pharmacy, supermarket, convenience store) using definitions from the North American Industry Classification System (NAICS) definitions, coded with supermarkets as the reference category [19]. *Total amount of tobacco marketing* material was derived from the store audit data and included counts of tobacco ads on the store interior and exterior, branded functional items (e.g. change mats), and tobacco moveable displays.

##### Neighborhood

We used census tracts as neighborhood. Using the latitude and longitude of the store taken at the front entrance, we linked store location to the following neighborhood characteristics: the percentage of black and Hispanic residents, derived from 2010 US census data [20]; and the percentage of families living below federal poverty guidelines, based on the 2006–2010 American Community Survey 5-year estimates [21].

##### County

Our prior study found that odds of compliance varied significantly by county [4].

### Data analysis

We calculated descriptive statistics to characterize the study sample and patterns of source of information about regulations, awareness of, and barriers to compliance with the Tobacco Control Act, support for POS policy, and compliance with POS policy. Confirmatory Factor Analysis (CFA) of the Barriers items indicated that the 4 items formed a unidimensional scale with excellent fit based on established cutoff criteria (*χ*^2^ =.92 df = 2 *p* = .63; RMSEA .00 90 % CI 0.00, .12 CFI = 1.00; TLI = 1.03; SRMR = .014) [22]. All four items loaded significantly onto the latent factor. For the Level of Support measure, CFA found that the items formed a unidimensional scale with excellent model fit (*χ*^2^ =10.97 df = 16 *p* = .81; RMSEA 0.0 90 % CI 0.0, 0.037; SRMR = .02; CFI = 1.000; TLI = 1.01). Residuals for each of the pairs of items for each POS domain were correlated. All items loaded significantly onto a single support for POS factor with the exception of the two minor’s access items which had over 90 % support and thus little variance. They were dropped from the scale for adjusted analyses.

We conducted bivariate analyses (not shown) using Fisher’s exact test and logistic regression for binary variables and ANOVA and Pearson correlations for continuous variables. Covariates that were significant at *p* < .05 were included in adjusted analyses. We conducted analyses looking at the relationships of Awareness, Source of information, and Barriers, and Support for POS policies with likelihood of noncompliance using GEE using PROC GENMOD in SAS 9.3. We used GEE because the intraclass correlation (ICC) of the null model using census tract as the clustering variable showed that 11 % of the variance in noncompliance was due to neighborhood (census tract). Because of this clustering, independence of stores cannot be assumed and GEE calculates robust standard errors using an exchangeable covariance structure to ensure appropriate confidence intervals. Additionally, we adjusted for sample weights at the county level to account for the sampling design.

For these analyses we separately modeled the factors associated with extent of change required (operationalized as awareness, source of information, and barriers) and retailer support for policies. These separate analyses were conducted to understand these facts as separate theoretical constructs. Empirically, barriers items were only asked of managers and owners but not of clerks, also supporting separate analyses.

We added covariates (not shown) in hierarchical sets [4]. We entered the control variables in order from the most ‘proximal’ to the most ‘distal’ influences: theory driven, individual, store-level, neighborhood, and then county-level factors. The final model with all hierarchical sets of variables had the best model fit based on the quasi-likelihood under the independence model criterion (QIC) [23].

## Results

### Sample description

Among the 252 stores in the study, 16 % were noncompliant with Tobacco Control Act provisions based on our store audits. The predominant type of violations shown in Table 1 were sales of modified risk labeled cigarettes (e.g., “light” or “low tar”) (13 % of stores) and self-service displays of cigarettes or smokeless tobacco (2 % of stores). Interview respondents, shown in Table 1, were predominantly store managers or assistant managers (54 %), followed by clerks (35 %) and owners (12 %). Smoking prevalence among respondents (some day or everyday) was 40 %, higher than the 21.8 % smoking rate in North Carolina in 2011 [24]. The predominant store type was gas station or gas station with convenience stores (53 %). On average, stores had 34 tobacco marketing materials and 16 % were within 1000 ft of a K-12 public school. Stores in the sample were in 116 neighborhoods (i.e., census tracts). On average, retailer neighborhoods were similar to the state as a whole with 21.8 % Black residents (vs. 21.5 % for the state) and 9.6 % (vs. 8.4 %) Hispanic residents, but had higher percentages of residents with a college degree (31.1 % vs. 26.1 %), and fewer residents under family poverty thresholds (12.5 % vs. 15.5 %) than the state.

**Table 1:** Descriptive characteristics of interview respondents

	Mean (SD) or n (%) (*n* = 252)
Awareness of FDA regulations (*n* = 248) (%)	106 (42.7)
Formal source of information (*n* = 248) (%)	241 (97.2)
Barriers (Owners/Managers *n* = 162) (Mean)	2.52 (0.9)
Support for POS regulations (*n* = 249) (Mean)	3.15 (0.7)
Noncompliant (*n* = 252) (%)	41 (16.3)
Types of violations (*n* = 252) (%)	
Sale of cigarettes claiming modified risk (labels such as “light” or “low tar”)	33 (13.1)
Self-service display for cigarettes or smokeless tobacco	5 (2.0 %)
Flavored cigarettes	1 (0.4)
Branded non-tobacco products	1 (0.4)
Sale of smokeless tobacco in less than full package	1 (0.4)
Individual characteristics	
Smoking status (*n* = 249)	
Never smokes (%)	149 (59.8)
Smokes every or some days (%)	100 (40.2)
Respondent Type	
Store owner (%)	29 (11.5)
Store manager (%)	135 (53.6)
Store clerk (%)	88 (34.9)
Store characteristics	
Store type	
Grocery store/supermarket (%)	40 (15.9)
Gas station/gas convenience (%)	134 (53.2)
Convenience (%)	33 (13.1)
Drug store/pharmacy (%)	26 (10.3)
Tobacco store (%)	11 (4.4)
Other store (%)	8 (3.2)
Number of tobacco marketing materials (Mean)	34.13 (SD 19.60)
Proximity to school	
Greater than 1000 ft. (%)	211 (83.7)
Within 1000 ft. (%)	41 (16.3)
Retailer neighborhood characteristics (Mean)	
% Black residents	21.8 (SD 22.3)
% Hispanic residents	9.6 (SD 8.7)
% Bachelors or More (%)	31.9 (SD 16.3)
% Family poverty	12.5 (SD 12.1)
County	
Durham (%)	79 (31.4)
Buncombe (%)	91 (36.1)
New Hanover (%)	82 (36.1)

We conducted analyses for non-response bias by examining bivariate relationships between respondents (*n* = 252) and non-respondents (*n* = 72) using chi-square tests for categorical variables and t-tests for continuous variables. These analyses showed no significant differences by any store, neighborhood or county characteristic among stores that completed the interview and those that did not participate (analyses not shown). Stores also did not differ on noncompliance between responders (16.3 %) and non-responders (16.8 %) (*χ*^2^ (1) = .0064 *p* = .94).

### Descriptive statistics

#### Awareness of the tobacco control act

Fewer than half of respondents (43 %) were aware of the Tobacco Control Act 3 years after its implementation.

#### Source of information about tobacco control regulations

**Fig. 2:**
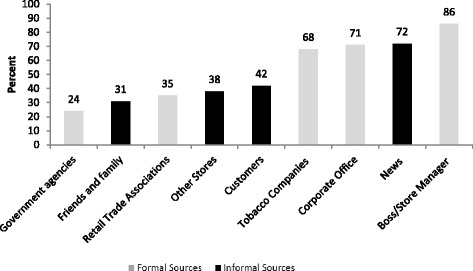
Percent of respondents citing category as a ‘usual source’ of Information about tobacco control regulations

Percent of respondents listing each source of information from those who answered yes or no to that source is shown in Fig. 2. Almost all respondents (97 %) had at least one formal source of information. The least common usual source of information about tobacco control regulations was government agencies (24 %). In contrast, boss/store managers were cited by 86 % of respondents; corporate offices for chain stores were also highly cited (71 %). Almost 70 % of retailers cited tobacco companies as a usual source of information about regulations.

#### Barriers

Overall, 41 % of owner and manager respondents noted at least one barrier to complying with regulations, with the most common that making changes to how tobacco is sold hurts their business (29 %). Less than one-quarter agreed that it was too hard to redo store space/displays (24 %), took too much time to make required changes (23 %), or was too costly (22 %).

#### Support for POS regulations

**Fig. 3:**
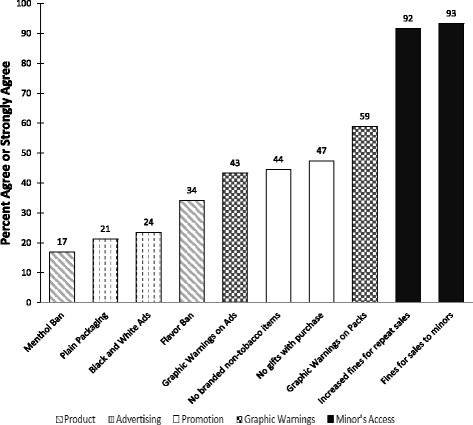
Percent agree or strongly agree with each POS provision (*n* = 252)

As shown in Fig. 3, respondents varied in the percent who agreed or strongly agreed with each particular POS provision. At least 90 % of respondents supported minor’s access provisions while the least support was found for a menthol ban, at 17 %. A large minority (over 40 %) support graphic warnings and promotion bans.

In looking at correlates of compliance, we ran GEE models for theoretical factors -- Awareness, Source of information, and Barriers, (Model A, Table 2) (*n* = 161 owners and managers only) and a separate model using Support for POS policies (Model B, Table 2) (*n* = 249). Awareness of regulations was significantly related to non-compliance only in model 3 when controlling for individual respondent and store covariates. Stores without formal sources of information about regulations were more likely to be non-compliant in models 2 and 3, including individual and store covariates. When adding neighborhood and county characteristics to the model, this result was no longer statistically significant. Sensitivity analyses using Fisher’s exact test (not shown) examining source of information by neighborhood demographic quintile found that lacking a formal source did significantly differ with the percent of black residents (*p* = .04) and with education level of residents (*p* = .03) in store neighborhoods. Lack of formal source appeared to be more common in areas with a lower percent of black residents and a lower percent of residents with a bachelor’s degree. In all models, retailers expressing higher levels of barriers had significantly higher odds of noncompliance. In model 5, accounting for individual, store, retailer neighborhood and county characteristics, stores with higher levels of barriers had 5.6 times the odds of non-compliance (AOR = 5.56, 95 % CI 2.24, 12.26). In model 5, stores in neighborhoods with more African-American residents had 8 % less likelihood of a violation (AOR = 0.92, 95 % CI 0.87, 0.97). But in neighborhood with more family poverty, there was 11 % increased likelihood of a violation (AOR: 1.11, 95 % CI 1.05, 1.18). No other covariates were significantly related to noncompliance (analyses not shown).

**Table 2:** Retailer opinions associated with store noncompliance with POS provisions

Constructs [AOR (95 % CI)] (*n* = 161)	Model 1 Barriers awareness and source	Model 2 Individual covariates	Model 3 Individual and store covariates	Model 4 Individual, store and neighborhood covariates	Model 5 Individual, store, neighborhood and county covariates
Model A (Owners/Managers *n* = 161)					
Awareness of regulations	1.99 (.84, 4.75)	2.03 (.83, 4.97)	**2.61 (1.14, 5.95)**	2.74 (.95, 7.78)	2.07 (.65, 6.58)
Source of information	.19 (.03, 1.15)	**.11 (.01, .88)**	**.06 (.01, .81)**	.03 (.00, 1.32)	.03 (.00, 1.45)
Barriers	**2.13 (1.31, 3.46)**	**2.42 (1.38, 4.25)**	**2.64 (1.49, 4.68)**	**4.62 (2.47, 8.64)**	**5.56 (2.24, 12.26)**
Model B (Owners/Managers/Clerks *n* = 249)					
POS support	**.61 (.37, 1.00)**	**.56 (.34, .90)**	**.57 (.33, .99)**	**.59 (.35, .99)**	**.59 (.36, .97)**

General Estimating Equations (GEE) controlling for covariates: Model 2 individual (smoking status, respondent type); Model 3 store (store type, total amount of tobacco marketing material); Model 4 neighborhood (% African American residents, % Hispanic residents, % residents under family poverty, % residents with college degree); and Model 5 county

Bold font indicates factors that are statistically significant at the *p* < .05 level

Table 2, Model B shows the results for using Support for POS policies as a correlate of noncompliance. In each model, greater support for POS provisions was associated with decreased odds of noncompliance. In the final model accounting for individual, store, neighborhood, and county covariates, for every one-unit increase in level of support for POS provisions, store likelihood of a violation decreased by 41 % (AOR = .59, 95 % CI: .36, .97). In this model, among covariates only pharmacies had higher odds of noncompliance compared with grocery stores (AOR = 3.43, 95 % CI: 1.05, 11.20). No other covariate was significantly related to noncompliance (not shown).

## Discussion

This study suggests that store noncompliance with FDA POS provisions is significantly related to both barriers and lack of support for POS provisions among retailers. However, compliance was unrelated to awareness of the Tobacco Control Act or having formal sources of information about tobacco control regulations.

In the final model, higher levels of barriers were positively associated with over 5 times the odds of noncompliance with Tobacco Control Act policies. This finding was consistent with the theoretical framework that the more change that the policy requires of implementers (operationalized as barriers), policy compliance is reduced [9]. However, this study could not assess the causal direction of this relationship. Findings about barriers are in line with prior studies of tobacco retailers indicating that barriers such as use of false identification made it difficult to comply with restricting sales to minors [25] and that lack of space is a barrier to displaying anti-tobacco messages [26].

Other authors have found that compliance with smokefree air [27] or minor’s access regulations [28] is related to awareness of regulations. In this study, awareness of the Tobacco Control Act among retailers was relatively low (43 %), but did not correspond with violations of POS provisions. However, the study was conducted in a period prior to FDA inspections of compliance with the Tobacco Control Act in North Carolina, which may increase both compliance and awareness over time. Moreover, in some cases retailers could be unknowingly compliant because their wholesaler stopped providing them with restricted products (e.g., no more flavored cigarettes, cigarettes without ‘lights’ descriptors).

Our study also found that having formal sources of information was unrelated to compliance once controlling for store neighborhood and county characteristics. Our results contradict a prior study, which found that compliance with smokefree air legislation at worksites was related to citing formal sources of information about regulations, rather than informal sources like friends or family [29]. Our null finding in models controlling for neighborhood and county characteristics may be related to the fact that most stores had a formal source of information about regulations. However, the fact that results were attenuated when controlling for neighborhood and county covariates perhaps suggests that some communities may differentially lack a formal source and may need more targeted outreach. Even so, in this study, the formal source least cited was government agencies (24 %), which are tasked with enforcement of these regulations. In contrast, almost 70 % of stores received information about regulations from tobacco companies, who have used prior retailer programs to build ties with retailers to undermine tobacco control efforts [30]. Boss/stores managers were the most cited source of information and may be a valuable conduit for relaying tobacco control information.

Support for policies was also significantly related to retailer compliance. A national telephone survey found that 66 % of retailers thought it should be illegal for retailers to sell tobacco to minors [25]. However, no prior study examined the relationship between retailer support for policy and compliance. We found higher support for provisions in the Tobacco Control Act that had been enacted the longest. Over 90 % of retailers supported minor’s access provisions enacted under the 1992 Synar Amendment, and over 40 % supported promotion restrictions implemented under the 1999 Master Settlement Agreement. For newer or proposed policies, less than a quarter to a third of respondents agreed with tobacco advertising restrictions or bans on flavored or menthol cigarettes.

The exception was relatively high levels of support among retailers for graphic warning labels on cigarette packs (59 %) and graphic warnings on advertisements in stores (43 %). Overall, the level of support among retailers for most of the provisions more closely resembled the level of support we found in a similar study among the general public (smokers and nonsmokers) than among smokers who had significantly lower support for every provision compared with non-smokers [31].

Overall, the findings from this study best support Mazmanian and Sabatier’s “Effective Implementation” scenario which describes a time of rapidly rising compliance after policy implementation followed by high levels of compliance maintained over time [8]. For the POS provisions that have already been implemented, compliance was relatively high even prior to enforcement. It is likely to improve further once active inspections, warnings, and fines for noncompliance begin. Additionally, as noted, support for enacted provisions was high among retailers, suggesting few barriers to implementation success over time. However, to see “effective implementation” with provisions that have not yet been implemented, will need more effort. Public health advocates would do well to work with retailers to improve support for these provisions and enhance the climate for implementation over time. Fruitful strategies may include direct inclusion of supportive retailers in policy advocacy action; engagement of retail trade associations or corporate chains; media campaigns aimed at tobacco control in the point-of-sale environment; or direct outreach, education, or retailer training activities (e.g., FDA’s Break the Chain retailer education program).

### Limitations and strengths

We note several limitations and strengths of the research. First, the temporal sequence of data collection of audit followed by interview, limits our ability to make causal inferences. We cannot determine whether more supportive retailers and those with fewer barriers are more likely to be compliant, or whether retailer compliance enhances retailer support and reduces perceived barriers. Nevertheless this research shows that these factors are associated over and above individual, store, neighborhood, and county characteristics. Future longitudinal studies are needed to separate out these effects. However, we do note that the research audits that were part of this study were not conducted for compliance or enforcement purposes. During the time period of the study, North Carolina had not yet been awarded a compliance and enforcement contract by the FDA. Thus, no enforcement inspections were active at the time of the study that may have influenced retailer compliance or attitudes toward regulations during the study period.

Second, the study was conducted in only three counties in North Carolina, which limits generalizability. Counties were selected to include diverse geographic areas of the state (a mountain, coastal, and central county of the state) and stores were randomly selected from a comprehensive list of stores within each county.

Measuring awareness of the Tobacco Control Act with only one item may have also been a limitation. Measuring awareness or knowledge of specific provisions as conducted in prior studies may have better correlated with compliance [28]. Additionally, there was little variance in the dichotomous measure of whether retailers had a formal source of information about tobacco control regulations. Additional research about retailers’ trust in different sources about tobacco control regulations may be more salient in improving compliance, as has been found in other areas [32]. Social desirability of responses is possible in an interviewer-administered survey, but appears unlikely as personal information about respondents was limited and they were not asked about compliance with regulations.

The study also has several strengths. It is one of the only studies that includes tobacco retailer opinion about policies and links it to observations of retailer compliance [28, 33] and the only one, thus far, which does so in relation to compliance with newer sales and marketing provisions of the Tobacco Control Act. It also uses theoretically derived factors that may influence policy implementation. Finally, it has a relatively large sample size and high response rate for retailer interviews.

## Conclusions

Understanding the relationship between retailer opinions and retailer compliance with POS provisions is important to helping implement Tobacco Control Act provisions. The Mazmanian and Sabatier framework would predict that policies that are ‘easier’ to implement or require few changes on the part of the retailer (e.g., passive retailer compliance due to changes in manufacturing or product availability) will have more compliance, while those that require active change (e.g., no sales or loose cigarettes, no self-service displays) will require more effort to gain compliance. Thus, helping retailers to address and overcome barriers, such as time and cost of implementing regulations, may enable them to become more compliant with these provisions. In most analyses, retailer awareness of the Tobacco Control Act and FDA authority over tobacco products was not necessary for them to comply with regulations. Instead guidance on specific provisions and how to successfully implement them as well as how to train staff to achieve compliance may be more valuable to retailers in overcoming barriers. This research also shows that few tobacco retailers were getting information about tobacco control regulations from government agencies. As such provisions are enforced, government agencies tasked with enforcement can do a better job communicating with and educating retailers about regulatory changes. Working through bosses and store managers and with small stores without corporate support can be a valuable approach to gaining support for tobacco control measures among retail staff who are ultimately responsible for implementing these policies.

This research also documents retailer support for specific POS measures. It is encouraging that some retailers are supportive of many POS policies. For proposed provisions with little support, advocates need to work with retailers to mitigate opposition to controversial provisions such as banning menthol cigarettes. While in some instances retail trade associations and some retailers have been opponents of tobacco control regulations and allies of the tobacco industry [2, 34], this research demonstrates that individual retailers have more varied opinions toward tobacco control regulations and may be engaged as stakeholders in tobacco control efforts at the POS.
